# Study of the UTMD-Based Delivery System to Induce Cervical Cancer Cell Apoptosis and Inhibit Proliferation with *shRNA targeting Survivin*

**DOI:** 10.3390/ijms14011763

**Published:** 2013-01-16

**Authors:** Zhi-Yi Chen, Kun Liang, Yan Lin, Feng Yang

**Affiliations:** 1 Department of Medical Ultrasound, Key Laboratory for Major Obstetric Diseases of Guangdong Province, the Third Affiliated Hospital of Guangzhou Medical University, Guangzhou 510150, China; E-Mails: linyan_cndmi@126.com (Y.L.); yangfeng_cndmi@126.com (F.Y.); 2 Guangzhou Research Institute of Obstetrics and Gynecology, Key Laboratory for Major Obstetric Diseases of Guangdong Province, the Third Affiliated Hospital of Guangzhou Medical University, Institute of Obstetrics and Gynecology, Guangzhou 510150, China; E-Mail: lk2008118@126.com

**Keywords:** ultrasound, microbubble, apoptosis, gene therapy, RNA interference, non-viral vector

## Abstract

Apoptosis induction by short hairpin RNA (shRNA) expression vectors could be an efficient and promising strategy for cancer gene therapy. Ultrasound-targeted microbubble destruction (UTMD) is an appealing technique. In this study, we investigated the apoptosis induction and suppression of cell proliferation *in vivo* transfected by the UTMD-based shRNA delivery system. Nude mice with transplanted tumors of cervical cancer were randomly arranged into three groups: control group, plasmid injection and ultrasound (P + US), P + UTMD group. Expressions of Survivin and proliferating cell nuclear antigen (PCNA), Bcl-2, Bax, Caspase-3, Ki-67, nucleostemin (NS) were investigated by immunohistochemistry. Furthermore, microvessel density (MVD) was detected by CD34 protein expressions and apoptotic index (AI) was measured by TUNEL. As compared with those in the control and P + US groups, protein expressions of PCNA, Ki-67, Bcl-2, Survivin and NS in P + UTMD groups were down-regulated markedly, while those of Bax, Caspase-3 were up-regulated significantly (*p* < 0.05). MVD decreased significantly, whereas AI increased remarkably (*p* < 0.05). We suggested that UTMD-based shRNA delivery system could induce apoptosis and inhibit proliferation significantly, without causing any apparently adverse effect, representing a new, promising technology that would be used in the future gene therapy and research.

## 1. Introduction

In recent years, gene silencing mediated by RNA interference (RNAi) technology shows therapeutic potential on solid cancer, and an in-human Phase I clinical trial that used a targeted nanoparticle-delivery system has been conducted [1]. Now, two types of RNAi-based therapeutics are available: the chemically synthesized double-stranded small interfering RNA (siRNA) or vector based short hairpin RNA (shRNA) [2]. Compared with other methods, shRNA is a better and more effective tool for cancer treatment.

For all the existence of the specific and effective gene silencing, the major hurdle for delivering RNA effectively to the target cell still remains. Lots of gene delivery systems such as cationic polymers or liposome exist, which are effective in transfecting cells *in vitro*. However, they cannot be used *in vivo* due to low target specificity. To achieve successful gene therapy, it is important that gene delivery systems be safe and easy to apply. Because of considerable immunogenicity related to use of viruses, non-viral gene transfer still needs to be developed. Recently, among non-viral gene transfer methods, it has been shown that ultrasound targeted microbubbles destruction (UTMD) increases cell membrane permeabilization and induces sonoporation, which has been explored to provide a safe and potentially effective approach for gene delivery.

There are a few articles that demonstrate sonoporation using microbubble promoted plasmid siRNA/shRNA transduction [3–8]. For potential use of UTMD as a therapeutic gene delivery system, it is critically important to investigate apoptosis induction under actual physiological conditions. Hence, xenograft tumors in nude mice are further investigated. However, there have been few reports regarding the transfection of shRNA into a targeted solid tumor using the UTMD-based delivery system *in vivo*. Solid tumor is the main cause of death. Although the anti-cancer drugs can effectively induce tumor cell death, their therapeutic effect *in vivo* is not so ideal [9]; this is related to nonuniform and insufficient identifiable medicine or therapeutic gene level in tumor microenvironment. Tumor vasculature and the extracellular matrix are the key influencing factors of drug or gene delivery.

Our previous study has already reported use of UTMD combined with polyethylenimine (PEI) could enhance targeted gene delivery and gene expression in tumor xenografts at intravenous administration effectively without causing any apparently adverse effect. We also observed that survivin gene could be regarded as a potential target for gene therapy in cervical cancer. Survivin-shRNA facilitated by UTMD technology could efficiently and specifically knock down surviving gene expression, induce cell apoptosis and inhibit proliferation in vivo significantly [6,7].

We have described a study of UTMD combined with shRNA technique. Survivin gene knocking-down using UTMD combined with shRNA can result in apoptosis induction and proliferation inhibition by evaluating the expressing levels of multiple apoptotic and anti-apoptotic markers. This current work is further analysis based in the previous study to evaluate the therapeutic value of this promising approach. We applied multiple assays to support the conclusion. In this study, UTMD-based system was applied to delivery shRNA targeting Survivin gene to suppress tumor growth within nude mice models transplanted with HeLa cells and to explore its feasibility and effectiveness. The results showed that UTMD-based shRNA delivery system could significantly induce apoptosis of transplanted tumor cells in nude mice, inhibit cell proliferation without any obvious side effects by evaluating the expression levels of multiple apoptotic and anti-apoptotic markers that were not studied previously. Moreover, microvessel density (MVD) and the apoptotic index (AI) were detected to support the conclusion. UTMD-based delivery system is an effective, noninvasive, promising new method for cancer gene therapy. This study concerns an interesting area of investigation and is compelling to researchers working in this field.

## 2. Results

### 2.1. Down-Regulation of Survivin Retarding Tumor Growth in Nude Mice

**Figure 1:**
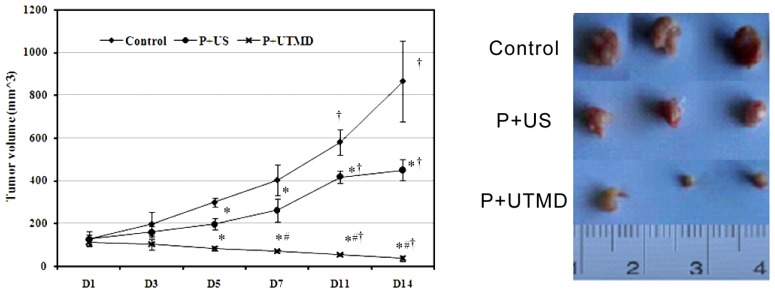
Comparison of the tumor volume in different groups. Control: control group; P + US: plasmid + ultrasound irradiation group; P + Ultrasound-targeted microbubble destruction (UTMD): plasmid + microbubble + ultrasound irradiation group; D1–D14: day 1–day 14; At the same time point, as compared with control group, * *p* < 0.05; as compared with P + US group, ^#^*p* < 0.01; as compared with D1, ^†^*p* < 0.01.

To explore possible effect of Survivin on tumor growth *in vivo*, tumor formation assay in nude mice was performed. As shown in Figure 1A, on the first day and third day after treatment, the difference of tumor volume in each group was not significant (*p* > 0.05). Seven days after treatment, the volumes of tumor sizes in P + UTMD group were decreased significantly (*p* = 0.031), while at the 11th and 14th day, the tumor volume was significantly reduced, and the difference was significant as compared with the first day and third day (all *p* < 0.05). After treatment, the tumor volume was still increased in P + US group and control group gradually. At the end of treatment, the tumor volumes in P + UTMD group were smaller than those of control group and P + US group significantly (*p* = 0.000, 0.010). As compared with control group, tumor volumes of P + US group were decreased significantly (*p* = 0.010). Average weight of transplanted tumor in P + UTMD group was significantly less than those in in control group and P + US group (*p* < 0.05). The inhibition rate was significantly higher than that of P + US group (72.20% ± 9.28% *vs.* 37.00% ± 4.33%), and the difference was significant (Figure 1B, *p* < 0.01). The data showed that UTMD-based shRNA delivery system could mediate down-regulation of Survivin expression exerted a potent growth inhibitory effect *in vivo*.

### 2.2. Immunohistochemical Findings *in Vivo*

Positive expression of Survivin, PCNA, Ki-67, Caspase-3, Bcl-2, Bax or NS protein are those significant yellow or brown particles were observed within tumor cells. Positive reactions of PCNA, Ki-67, NS were mainly located in the nucleus, while Survivin, Caspase-3, Bax and Bcl-2 positive reaction were mainly located in intracytoplasm.

**Figure 2:**
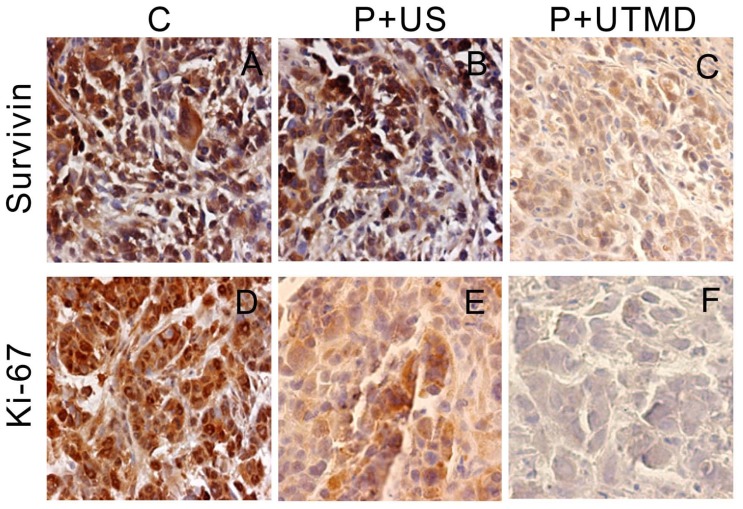

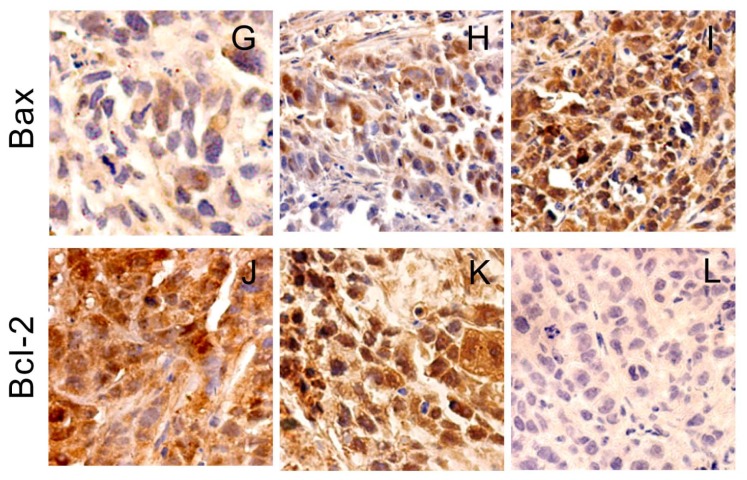
Expression of Survivin, Ki-67, Bax and Bcl-2 of tumor specimens in xenografts (400×). C: control group; P + US: plasmid + ultrasound irradiation group; P + UTMD: plasmid + microbubble + ultrasound irradiation group.

The scores of Survivin expression in control group (4.33 ± 0.69) and P+US group (4.11 ± 0.67) were higher than that of UTMD group (1.27 ± 0.57) significantly (both *p* < 0.01). Scores of Ki-67, PCNA protein expression of control group were (4.06 ± 0.73) and (3.83 ± 0.62), and that of (P + US group were (3.22 ± 0.88) and (3.39 ± 0.61). In P + UTMD group, scores of Ki-67, PCNA protein expression of were (1.22 ± 0.43) and (1.56 ± 0.51), respectively. As compared with P + US group and control group, the difference was significant (*p* < 0.01). As showed in Figure 2, some part of the tumor sample displayed no Survivin or Ki-67 protein expression, indicating that UTMD-based shRNA delivery system could effectively silence the Survivin gene expression and inhibit cell proliferation.

Compared with control group and P + US group, a lower number of Bcl-2 positive cells was observed in P + UTMD group (score, 4.17 ± 0.62 *vs.* 3.72 ± 0.57 *vs.* 1.94 ± 0.64, all *p* < 0.01). Moreover, score of Bax protein expressions in P + UTMD group was upregulated remarkably as compared with control group and P + US group (4.22 ± 0.65 *vs.* 2.22±0.65 *vs.* 1.61 ± 0.70, all *p* < 0.01). Moreover, little caspase-3 positive cells were showed in control group, and score of P + UTMD group was significantly increased (score, 1.56 ± 0.51 *vs.* 4.33 ± 0.59, *p* < 0.01). The results indicated that inhibition of Survivin by UTMD-based shRNA delivery system resulted in apoptosis induction by downregulating Bcl-2 expression and upregulating the activity of caspase-3 and Bax.

**Figure 3:**
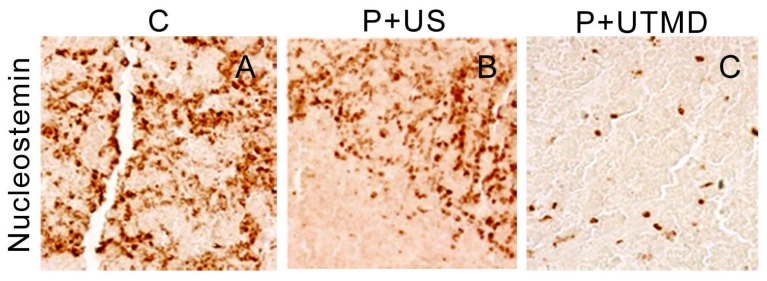
Comparison of NS gene expressions in different groups (×200). C: control group; P + US: plasmid + ultrasound irradiation group; P + UTMD: plasmid + microbubble + ultrasound irradiation group.

As shown in Figure 3, NS gene was highly expressed in the control group (3.89 ± 0.47), and expression was slightly reduced after treatment with P + US (3.44 ± 0.51). Silence of Survivin gene mediated by UTMD resulted in down-regulation of NS gene expression significantly, score was significantly decreased than that of the control group and the P + US group (1.38 ± 0.50), and the differences were statistically significant (both *p* < 0.01).

### 2.3. Microvessel Density

**Figure 4:**
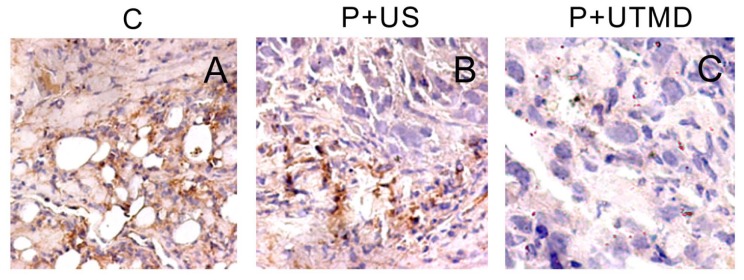
Comparison of CD34 expression in different groups (×400). C: control group; P + US: plasmid + ultrasound irradiation group; P + UTMD: plasmid + microbubble + ultrasound irradiation group.

CD34 was located in cell membrane or cytoplasm of microvascular endothelial cells. Appearance of CD34-tagged capillary was irregular and its distribution was uneven, which concentrated mainly at the edge of tumor invasion (Figure 4). Stronger CD34 expression in the control group (59.33 ± 9.07) indicating high MVD which was significantly higher than that of P + US group (35.33 ± 3.21) and P + UTMD group (10.33 ± 2.08), and the differences were statistically significant (*p* = 0.005, 0.000). But MVD of P + UTMD group was decreased than that of P + US group significantly (*p* = 0.004).

### 2.4. H & E Staining

**Figure 5:**
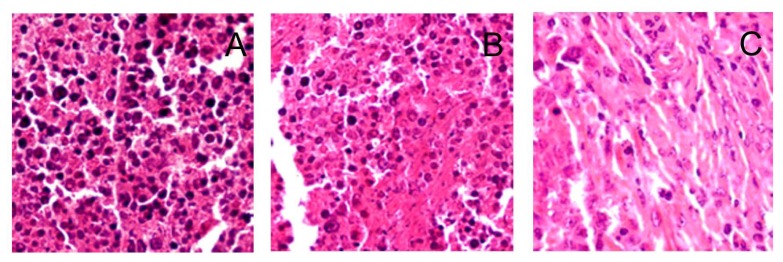

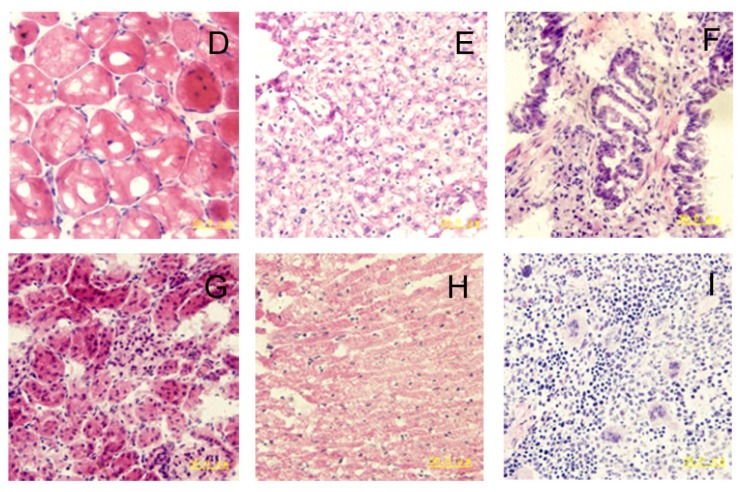
Expression of H & E in different groups. **A**: control group, ×200; **B**: plasmid + ultrasound group, ×200; **C**–I: plasmid + microbubble + ultrasound group, ×400; **A**–**C**: Transplanted tumor; **D**: muscle; **E**: liver; **F**: lung; **G**: kidney; **H**: heart; **I**: pancreas.

H & E staining showed that (Figure 5A–C), in the control group, tumor cells that had large, hyperchromatic and irregular nucleuses that were arranged in masses, with large nuclear-cytoplasmic ratios. A small number of the tumor cells experienced necrosis, and an increasing number of karyokinesises were visible. In the P + US and P + UTMD group, the tumor cells became smaller, apoptotic bodies were appeared but karyokinesis rarely was observed. The heart, liver, muscle, pancreas, lung, kidney in P + UTMD groups underwent histological examination (Figure 5D–I). The results showed that the samples maintained good integrity and had no infection. No inflammatory cell infiltration, hemorrhage or edema was observed.

### 2.5. TUNEL Assay

**Figure 6:**
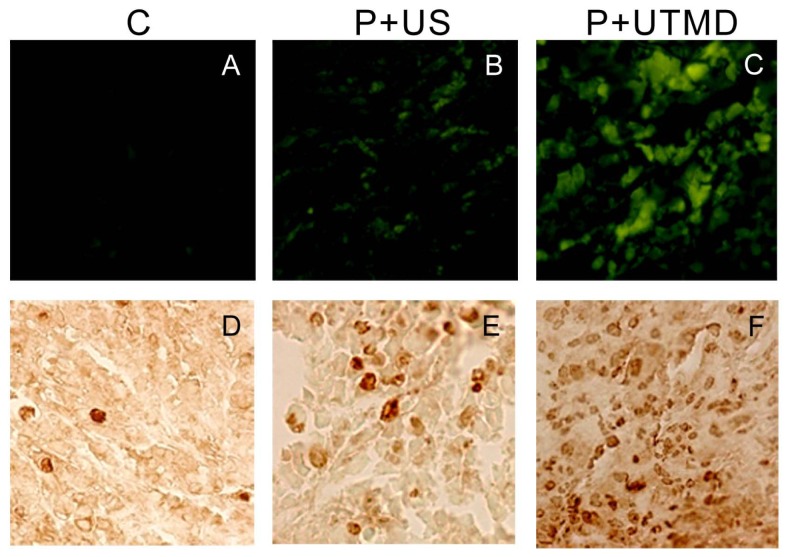
Detection of TUNEL expressions in different groups (400×). C: control group; P + US: plasmid + ultrasound irradiation group; P + UTMD: plasmid + microbubble + ultrasound irradiation group, **A**–**C**: Tunel assay; **D**–**F**: DAB detection.

AI of the tumor samples in P + UTMD group (29.07% ± 2.38%) were obviously higher than that of control group (1.70% ± 0.66%) and P + US group (7.27% ± 1.04%), and the differences were significant (Figure 6, *p* < 0.01).

## 3. Materials and Methods

### 3.1. Cell Culture

Human cervical cancer cell lines (HeLa) were obtained from China Center for Type Culture Collection (CCTCC) and incubated in Dulbecco’s modified Eagle’s medium (DMEM, Gibco, Invitrogen Corporation, Grand Island, NY, USA) with 10% fetal bovine serum (FBS, Gibco, Invitrogen Corporation, Grand Island, NY, USA) and 100 U/mL penicillin, 100 μg/mL streptomycin, at 37 °C in a humidified environment of 5% CO_2_ and 95% air. Total cell count was determined with a hemocytometer (Burker Turk). Initial cell viability was determined by means of exclusion with trypan blue dye (Sigma-Aldrich Corp. St. Louis, MO, USA).

### 3.2. shRNA Design and Plasmid Construction

shRNA expression vector targeting human survivin gene was designed and synthesized as described previously [6]. The specific recombinant shRNA vector was named survivin-shRNA. The selected reconstructed plasmid was extracted and purified using an endoFree plasmid maxi kit (Qiagen, Crawley, UK). The concentration of isolated plasmid DNA was determined and resuspended to a final concentration of 1 μg/μL in buffer.

### 3.3. Preparation of shRNA-Microbubble Complexes

The suspension of SonoVue^®^ microbubbles (Bracco Research, Geneva,, Switzerland) were reconstituted before use by injecting 5 mL of 0.9% saline solution according to the manufacturer’s protocol. SonoVue^®^ contained 2 × 10^8^–5 × 10^8^ microbubbles/mL and the shell is composed of phospholipid and encapsulates sulfur hexafluoride gas. Microbubble diameter is typically 1–10 μm. Before the experiments, plasmid DNA (30 μg/mouse) and SonoVue^®^ microbubble (100 μm, 11.8 μg/μL) were gently agitated with phosphate buffered saline (PBS) to a final volume of 200 μL to prepare the transfection complexes, and then they were transferred to the polystyrene tube and incubated at room temperature for 30 min. The total dose of injection was 200 μL.

### 3.4. Experimental Grouping of Gene Delivery

To analyze the impact of apoptosis induction and proliferation inhibition, nude mice bearing tumor xenografts were selected, randomly divided into three groups, six mice each group: control group (PBS), plasmid injection and ultrasound (P + US group), P + UTMD group.

### 3.5. Experiment Protocol

Female Balb/c (nu/nu) mice, 4–6 week old, weighing 15–21 g, were purchased from experimental animal research center. The animal study protocols were conducted according to approved institutional guidelines for animal use. The mice were inoculated subcutaneously into the flank with 2 × 10^6^ Hela cells per mouse after local sterilized. Animal modeling was prepared using methods described previously [6]. The mice were raised at specified pathogen free (SPF) qualification after operation, being observed one time every two days. Two weeks later, the experiments were initiated when the tumors reached a size of 5–10 mm.

The mice were anesthetized by diethylether and fixed on the flats. All the plasmid DNA or complexes were administrated by tail vein. After the injection, the tumor xenografts were sonicated immediatly by a therapeutic ultrasound transducer with surface area of 0.8 cm^2^ (Accusonic, Metron Medical Australia Pty. Ltd., Carrum Downs Victoria, Australia) placed on the skin with contact gel (Aquasonic 100, Parker Laboratories Inc., Fairfield, NJ, USA). Ultrasound parameters were set at 3 MHz, 2 W/cm^2^, 2 min, duty cycle 20% (*i.e.*, 2 ms “on” time and 8 ms “off” time). After inoculation, animals were observed every day, tumor size was determined by measuring two diameters perpendicular to each other with a caliper at 3, 5, 7, 11, 14 days. Tumor volume was calculated according to the following equation: *V* (mm^3^) = width^2^ (mm^2^) × length (mm)/2. Tumor volumes were measured weekly with an electronic caliper. Tumor growth curve was based on tumor volume. Inhibition rate = (tumor weight in control group − tumor weight in treatment group)/tumor weight in control group × 100%.

14 days after ultrasound treatment, animals were humanely sacrificed, tumor masses were rapidly removed and weighed, then fixed in 10% formalin, embedded in paraffin, and subjected to H & E or immunohistochemical staining.

### 3.6. Histopathology

Serial sections of tumor tissue were processed for histological examination. The specimens were washed with PBS to remove blood, fixed with formaldehyde, dehydrated with a graded alcohol series, and embedded in paraffin. Hematoxylin eosin staining (H & E) was performed on the specimens, for histopathologic evaluation of hemorrhage, necrosis, and inflammation.

### 3.7. Immunohistochemistry

The samples were fixed with formaldehyde, dehydrated with a graded alcohol series, and embedded in paraffin. The sections were incubated with primary antibodies against Survivin, Proliferating cell nuclear antigen (PCNA), Ki-67, Caspase-3, Bcl-2, Bax and Nucleostemin (NS) (1:100 dilution, all purchased from Santa Cruz Biotechnology) and then incubated with appropriate biotinylated secondary antibody. The colorimetric detection was performed by using a diaminobenzidine detection kit (Boster Biological Technology Co. Ltd., Wuhan, China). Images were acquired with a microscope (BX51, Olympus, Tokyo, Japan). The assessment of the immunohistochemical results were modified from that described previously [10,11].

Six slices were randomly selected as sample for semi-quantitative expression grading by three observers in order to achieve blinding observation. Only the scores that the observers agree to are used for analysis. The percentage of cells expressing the marker were classified qualitatively based on the intensity of staining and the percent of cells as follows [10,11]: score 1: no reactivity; score 2: low intensity staining in less than 10% of cells; score 3: low to moderate intensity staining in 11% to 30%; score 4: moderate to strong staining in 31% to 50%; score 5: diffuse, strong intensity staining in 50%.

### 3.8. Dection of Microvessel Density

By counting microvessels, angiogenesis can be quantified as an angiogenesis index, defined as MVD. Different endothelial markers, including CD31, CD34 and vWF, are used for staining vascular endothelial cells. As CD34 is the most sensitive of the three investigated markers for angiogenesis in cancer [12], the slides of the tumor tissues were incubated sequentially with CD34 polyclonal antibody (1:200; New Mark Co. Ltd., Kidlington, Oxford, UK), biotinylated anti-IgG antibody (1:300; Sigma-Aldrich Corp. St. Louis, MO, USA) and streptavidin-biotinylated-complex/horseradish peroxidase (Sigma). The sections were counterstained with hematoxylin andevaluated independently by two blinded investigators.

MVD is the average of the vessel counts obtained in the three sections. The areas representing the highest neovascularization were chosen, and microvessel counting was performed at 200× magnification in three chosen fields. Any immunoreactive endothelial cell or endothelial cell cluster that was separated distinctly from adjacent microvessels was considered a single countable vessel. The results regarding angiogenesis in each tumor were expressed as the absolute number of vessels/200× magnification field.

### 3.9. TUNEL Assay

TdT-mediated dUTP nick end labeling (TUNEL) staining was carried out using the cell apoptosis detection kit (Roche, Applied science, Indianapolis, IN, USA) according to the manufacturer’s instructions. The tissues present obvious apoptotic cells were regarded as positive control, and the reagent 2 (deoxyribonucleic acid mixture) without terminal TdT instead of TUNEL reaction mixture was regarded as negative control. We used light microscope to select 400× magnification fields randomly in the strongest immune response region for each tissue sections. Nuclei with clear brown staining were regarded as positive. The positive cells and total cells were counted. We identified that *AI* = number of apoptotic cells/total number of cells.

### 3.10. Statistical Analysis

Statistical analyses were performed by the SPSS 13.0 software package (SPSS, Inc, Chicago, IL, USA). All values were expressed as mean ± SD. Analysis of variance with *t* test and analysis of variance (ANOVA) test were used to determine the significance of the difference in a multiple comparison. If the ANOVA was significant, the Tukey’s procedure was used as a post hoc test. Differences with a *p* value of less than 0.05 were considered to be statistically significant.

## 4. Discussion

Since it is a challenge to efficiently deliver shRNA plasmid into tumor tissues in animals, in this study, we used UTMD to enhance the targeted delivery of Survivin-shRNA. When mixtures of microbubbles with shRNA plasmids were injected into tail vein of mice, the shRNA-microbubble complexes would circulate in the blood. When applied to the region of implanted tumor cells (*i.e.*, HeLa in this study), ultrasound would destroy microbubbles and perhaps trap plasmids in the tumor tissues, resulting in a targeted delivery of shRNA plasmids into tumor tissues. The current study was comprised of two concepts: silencing Survivin gene expression to repress tumor growth in a mouse model, and enabling targeted delivery of shRNA to tumor tissues by using UTMD. Although the concepts have been tested by many studies [6–8], different expressions of multiple apoptotic and anti-apoptotic markers were not studied previously. Moreover, this study attempted to use UTMD to change the tumor microenvironment, and the results indicated that, UTMD could effectively achieve uniform and adequate gene transfer, thereby resolving the problem of local gene delivery into the targeted tumor.

Survivin is an ideal target for cancer gene therapy. Survivin gene is essential to the growth of tumor cells in culture and *in vivo* as shown by numerous studies [13–15]. RNAi is an important down-regulation technology of specific Survivin gene expression. It produces a marked effect through directly inhibiting the activity of molecule Caspase-3 in the downstream of apoptosis signal pathway [13]. Caldas *et al.* [14] showed that application of the RNAi targeting Survivin could cure malignant tumors that highly express Survivin, causing apoptosis and micrangium degeneration. Zhang *et al.* [15] showed that intratumoral injection of adenovirus-delivered Survivin shRNA suppressed tumor growth by spontaneous apoptosis of cancer cells and significantly prolonged animal survival. In this study, we used RNAi as a tool to silence expression of survivin in nude mice bearing HeLa cells and then tested the therapeutic effects of silencing Survivin gene expression by RNAi achieved through shRNA plasmid. ShRNA-Survivin gene could effectively inhibit cell proliferation and promote apoptosis.

However, anti-cancer genes could not be completely transported to the tumor cells. Compared with normal tissues, tumor vascular permeability is larger, and there was greater interstice between endothelial cells [16]. Both spatial distribution of tumor vasculature and length and diameter of the capillary are irregular, which causes abnormal function of the vascular system [17]. Proliferative tumor cells can oppress blood vessels, thereby affecting their blood stream distribution. Another reason is the average distance between tumor and the blood vessels is farther, which reduces oxygen delivery and forms a hypoxic environment, resulting in accumulation of metabolites, limiting anti-cancer genes to reach the tumor cells far away, thereby reducing the curative effect [9]. On the other hand, extracellular matrix of cancer cells further limits the gene delivery [17]. McGuire [18] pointed out that, the larger collagen within the extracellular matrix of tumor cells, the higher perfusion pressure required to start the flow within tumor interstitium of drug or gene. Currently, strategies to strengthen tumor local delivery are being developed, such as the use of external energy [19].

UTMD is a new non-viral gene delivery method [20–22]. It is the incorporation of both ultrasound and microbubbles, which makes the UTMD system. Ultrasound itself can promote gene transfection and the destruction of microbubbles carrying genes can significantly increase gene transfection efficiency. SonoVue^®^ was widely used in clinic, which is an aqueous suspension of stabilized sulfur hexafluoride microbubbles. In the gene delivery system, therapeutic gene could be simply and non-invasively located in specific tissues or organs by ultrasound, which is safer and more effective compared with other methods. Tomizawa *et al.* [23] reported that ultrasound irradiation could increase the sensitivity of tumor tissues to bleomycin. Nie *et al.* [24] found that, compared to use of ultrasound irradiation alone, the tumor growth in ultrasound combined with SonoVue^®^ group was significantly inhibited, with the average life span of mice was prolonged, the expression of TK mRNA within the tumor increased, AI of tumor cells increased too. UTMD can effectively increase membrane permeability and promote specific drug or gene delivery.

Apoptosis can be spontaneous or induced by many factors [25]. Apoptosis induction provides an effective and promising cancer treatment. Studies [26–28] showed that, low intensity ultrasound also could induce apoptosis under certain *in vivo/in vitro* conditions. Honda *et al.* [26] pointed out that ultrasound induced apoptosis through the mitochondrial-Caspase pathway. On the other hand, apoptosis could be induced by transporting related genes through promoting the intracellular plasmid DNA delivery using ultrasonic energy [29]. Iwanaga *et al*. [30] found that, the growth of Ca9-22 cancer cells were significantly inhibited when microbubbles were insonated after being added into a bleomycins solution, while no apoptosis was detected when ultrasound or UTMD was applied separately. Duvshani-Eshet *et al.* [20] reported that, compared to ultrasound + plasmid group, the addition of ultrasound microbubbles was more conducive in increasing the target gene transfection, significantly reducing cell proliferation and migration in prostate cancer, and causing a significant increase in apoptosis.

The IAP family plays an important role in the regulation of apoptosis. In this study, we use the UTMD-based shRNA delivery system to perform a histological experiment and observe its influence on apoptosis and proliferation. Results showed that UTMD could achieve a high level of transgene activity within the target organs. And apoptosis induction and proliferation inhibition were located on the internal transplanted tumors, which received ultrasound irradiation, and maintained great sample integrity with no infection. The expressions of PCNA, Ki-67, Bcl-2, Survivin, NS protein in tumor samples were significantly decreased in P + UTMD group, while those of Bax and Caspase-3 were up-regulated significantly. MVD decreased significantly, whereas AI increased remarkably.

Bcl-2 is a suppressor gene of apoptosis. It can resist apoptosis induced by many factors, and enhance cell viability without affecting cell proliferation [31]. It also participates in the homeostasis regulation of apoptosis. Bax is an apoptosis activating gene, and it can form a heterodimer with Bcl-2 protein, and inhibit the function of Bcl-2 to promote cell apoptosis. Because Survivin is known to directly interact with caspase-3 and subsequently inhibit its activity, we analyzed caspase-3 expression in tumor sections. Microscopic examination of caspase-3 staining showed fewer positive cells in control group compared with UTMD group. UTMD-based shRNA delivery system can effectively start apoptosis induced by Caspase-3, and form a positive circulation with Bax, Bcl-2.

The results showed that, NS gene was highly expressed in the control group, which related with proliferation, development and metastasis of tumor cells. Survivin-shRNA transfected by UTMD delivery system could induce a significant down-regulation of NS gene expression to induce apoptosis, inhibit proliferation and promote differentiation. Moreover, we observed its moderate inhibitory effects on cell proliferation and tumor angiogenesis by CD34 staining, enhancing levels of caspase-3 protein and placing strong enhancing effect on apoptosis by TUNEL staining.

Higher efficiency of site-specific non-viral gene delivery is required to achieve therapeutic effects in clinical practice. In this view, UTMD is a feasible and effective tool to carry out gene therapy to treat diseases. However, many aspects of this experiment still need further studies, such as quantitative analysis of gene expression using better methods, and development of specific microbubbles and vectors to enhance gene transfection rate.

## 5. Conclusions

UTMD-based shRNA delivery system established in this work could specifically induce apoptosis in cervical cancer cells, inhibit proliferation *in vivo* and provide a powerful tool for gene function analysis. It also offers an efficient and safe new method for cancer gene therapy.
